# Supplementary material for: The Potential Role of an Aberrant Mucosal Immune Response to SARS-CoV-2 in the Pathogenesis of IgA Nephropathy
Source: Pathogens. 2021 Jul 12;10(7):881. doi: 10.3390/pathogens10070881 (PMC8308514; doi:10.3390/pathogens10070881)
Supplement: Supplementary file 1 [file pathogens-10-00881-s001.zip › pathogens-1227422-supplementary.pdf]

pathogens

MDPI

Supplementary Materials

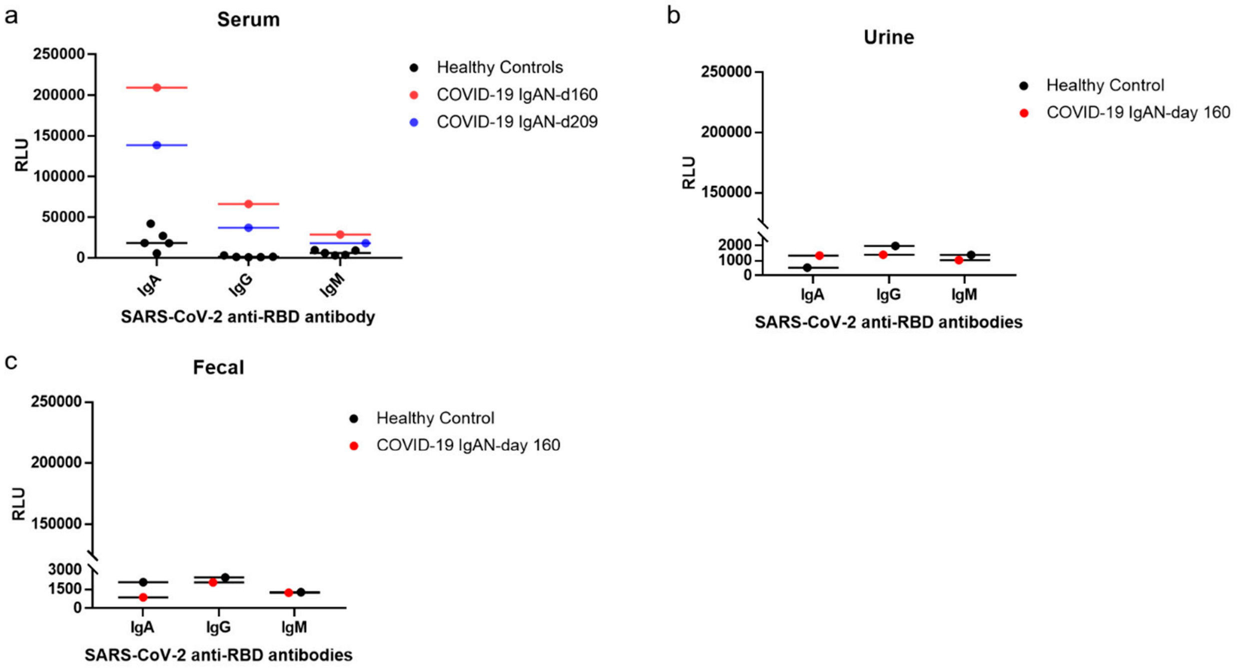

Figure S1. anti-SARS-CoV-2 spike protein RBD IgA, IgG, and IgM antibody concentrations in the serum, urine, and feces. **a.** IgA antibody levels remained high, even at seven months post-infection (anti-spike protein RBD IgA RLU: 138475±26834; anti-spike protein RBD IgM RLU: 18084±967; anti-spike protein RBD IgG RLU: 36991±7665). **b & c.** no increase in anti-SARS-CoV-2 spike protein RBD IgA, IgG, or IgM antibodies was observed in the urine or fecal samples of this COVID-19 IgAN case.
